# Development of a rabies virus-based retrograde tracer with high trans-monosynaptic efficiency by reshuffling glycoprotein

**DOI:** 10.1186/s13041-021-00821-7

**Published:** 2021-07-08

**Authors:** Fan Jia, Li Li, Haizhou Liu, Pei Lv, Xiangwei Shi, Yang Wu, Chen Ling, Fuqiang Xu

**Affiliations:** 1 Guangdong Provincial Key Laboratory of Brain Connectome and Behavior, CAS Key Laboratory of Brain Connectome and Manipulation, The Brain Cognition and Brain Disease Institute (BCBDI), Translational Research Center for the Nervous System (TRCNS), Shenzhen Institute of Advanced Technology, Chinese Academy of Sciences, Shenzhen, 518055 China; 2 Shenzhen-Hong Kong Institute of Brain Science-Shenzhen Fundamental Research Institutions, Shenzhen, 518055 China; 3 NMPA Key Laboratory for Research and Evaluation of Viral Vector Technology in Cell and Gene Therapy Medicinal Products, Key Laboratory of Quality Control Technology for Virus-Based Therapeutics, Guangdong Provincial Medical Products Administration, Shenzhen Key Laboratory of Viral Vectors for Biomedicine, Shenzhen Institute of Advanced Technology, Chinese Academy of Sciences, Shenzhen, 518055 China; 4 State Key Laboratory of Magnetic Resonance and Atomic and Molecular Physics, Key Laboratory of Magnetic Resonance in Biological Systems,, Wuhan Center for Magnetic Resonance, Innovation Academy for Precision Measurement Science and Technology, Chinese Academy of Sciences, Wuhan, 430071 China; 5 Wuhan National Laboratory for Optoelectronics, Huazhong University of Science and Technology, Wuhan, 430074 China; 6 University of Chinese Academy of Sciences, Beijing, 100049 China; 7 Wuhan Institute of Virology, Chinese Academy of Sciences, Wuhan, 430071 China; 8 Center for Excellence in Brain Science and Intelligence Technology, Chinese Academy of Sciences, Shanghai, 200031 China; 9 Division of Molecular and Cellular Therapy, Department of Pediatrics, College of Medicine, University of Florida, Gainesville, FL USA

## Abstract

Rabies virus (RV) is the most widely used vector for mapping neural circuits. Previous studies have shown that the RV glycoprotein can be a target to improve the retrograde transsynaptic tracing efficiency. However, the current versions still label only a small portion of all presynaptic neurons. Here, we reshuffled the oG sequence, a chimeric glycoprotein, with positive codon pair bias score (CPBS) based on bioinformatic analysis of mouse codon pair bias, generating ooG, a further optimized glycoprotein. Our experimental data reveal that the ooG has a higher expression level than the oG in vivo, which significantly increases the tracing efficiency by up to 12.6 and 62.1-fold compared to oG and B19G, respectively. The new tool can be used for labeling neural circuits Therefore, the approach reported here provides a convenient, efficient and universal strategy to improve protein expression for various application scenarios such as trans-synaptic tracing efficiency, cell engineering, and vaccine and oncolytic virus designs.

**Supplementary Information:**

The online version contains supplementary material available at 10.1186/s13041-021-00821-7.

## Introduction

Mapping neural circuits is a prerequisite to elucidate the mechanisms of brain functions. Several neurotropic viruses can infect neurons and spread across synapses between neurons, playing important roles in depicting the neurocircuit [1]. Among these virus-based tools, rabies virus (RV), encoding five proteins (N, P, M, G and L), is the most popular trans-monosynaptic viral tool to map the direct input networks of specific types of neurons in specific brain regions [2–6]. To realize cell type specific and trans-monosynaptic tracing, (i) the RV-G gene is deleted from genome (RV-delG); (ii) a chimeric EnvA gene is created by swapping the cytoplasmic part of G and wild type EnvA (a glycoprotein from ASLV-A; (iii) a cell line expressing the chimeric EnvA is prepared to package the genome of RV-delG to produce the pseudo-typed virus (EnvA-RV-delG); (iv) two adeno-associated virus (AAV) vectors (AAV-G, and AAV-TVA-GFP), cre-dependently delivering G-gene, GFP, and TVA (a receptor of EnvA), are constructed separately; (v) EnvA-RV-delG can enter specific neurons infected by AAV-TVA-GFP; (vi) in the neurons co-infected by EnvA-RV-delG/AAV-G/AAV-TVA-GFP, the G protein trans-complements the package of RVG-RV-delG which is capable of trans-monosynaptically retrograde spreading to input neurons [2, 3]. However, current RV versions can only label a fraction of presynaptic neurons [7]. When AAV-G infects neurons, the G expression cassette cannot be replicated because of the AAV features (or the template number for G-gene is fixed), while EnvA-RV-delG enters the same neuron, the G-deleted RV genome can be reproduced and expressed according to the RV life cycle.

Therefore, a significant mismatch between the amounts of G protein and other RV proteins might exist, leading to few active RV particles and thus low tracing efficiency, at the same time, generating excessive unused viral proteins and thus high cytotoxicity. Conventional methods for improving the protein expression level are to use stronger promoters, optimize the gene sequence, or provide more copies of the gene [8–13]. Indeed, previous reports have provided evidence that elevating the expression level of the G protein improves the tracing efficiency [14, 15]. The protein synthesis machinery has a preference for how to better translate proteins in different species. Codon pair bias (CPB) is a common phenomenon in various species for adjacent codon pairs, which appears with different frequencies in different species [16]. The favorite or unfavorite of a codon pair can be defined by its codon pair bias score (CPBS), defined as the ratio of observed frequency to the expected frequency. Every codon pair has a CPBS. A positive CPBS means that a codon pair is overrepresented, which may be preferred by the organism, whereas a negative CPBS may be unfavorable. Therefore, recoding a gene with positive CPBS may adjust protein expression. Many viruses have been attenuated by using the negative CPBS which results in decreased protein production [17–19], while only limited number of studies explored the effect of positive CPBS on viral properties. RV as a retrograde trans-monosynaptic tracer is most commonly used to label neural circuits in mice, however, mice are not the reservoir host. This cross-species shift may have a profound impact on the expression of proteins encoded by RV. The amount of G is a limiting factor for trans-synaptic efficiency of RV. Here, we propose that better conditions to increase the G protein level could be achieved by using positive CPBS in mice. Indeed, this convenient method can elevate the G expression level and improve RV trans-monosynaptic efficiency.

## Results

### The relationship of canine, mouse and human in CPB

**Fig. 1:**
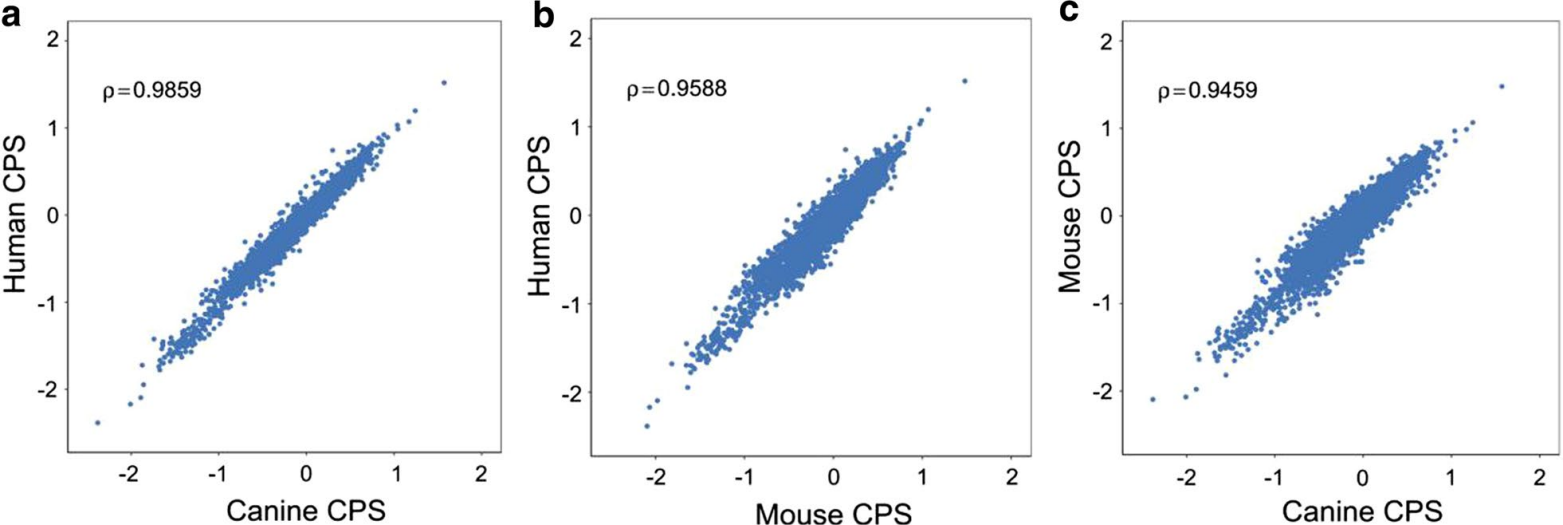
Codon pair bias in three speices (human, canine and mouse). **a**–**c** Each steel blue dot represents one of the 3721 possible codon pairs and shows codon pair score (CPS) in the human, the canine and the mouse. The CPSs of these species were separately calculated using the available genes of the human (110,788) (**a**), the canine (45,094) (**b**), and the mouse (68,272) (**c**) using a previous described method [17]

**Fig. 2:**
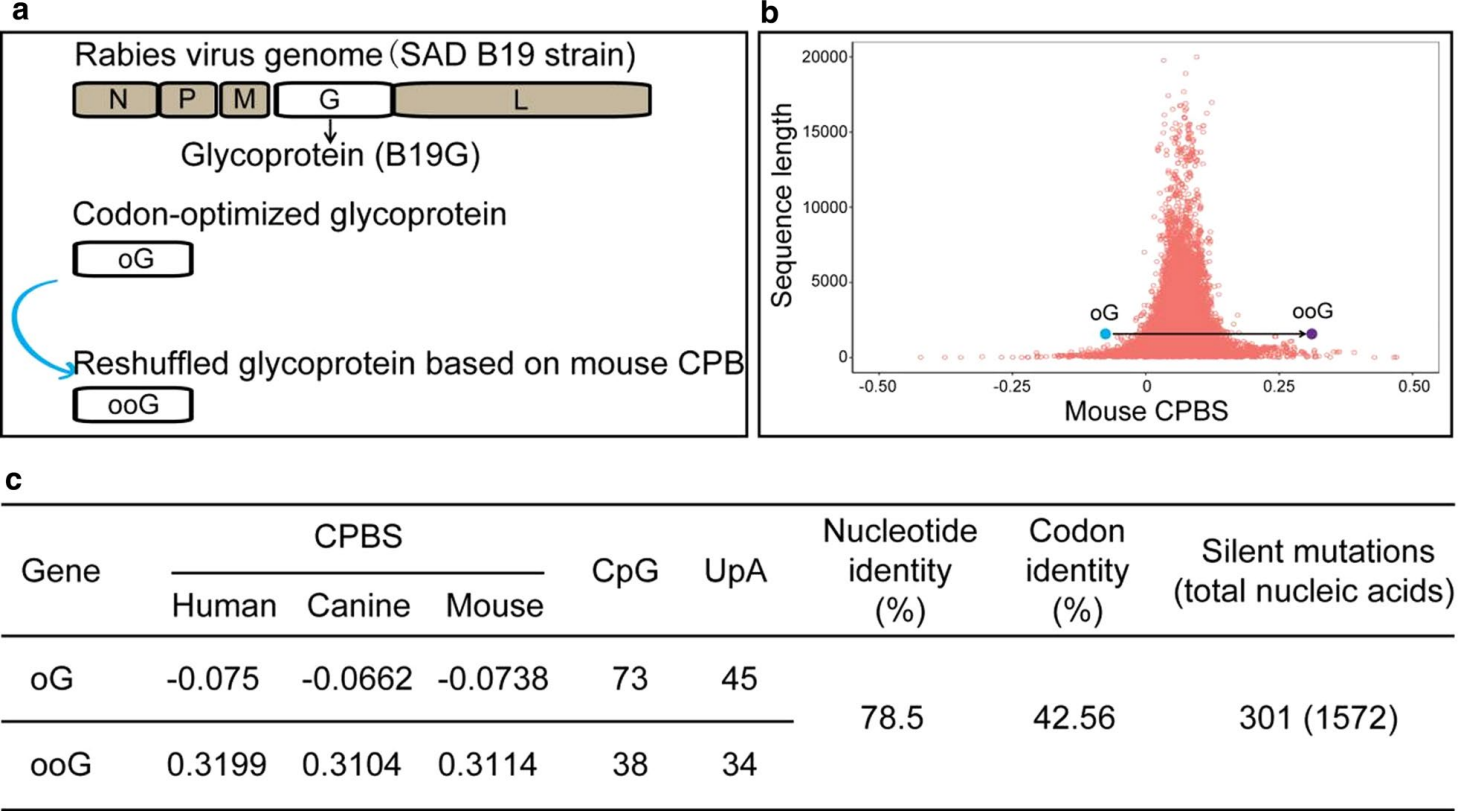
Reshuffling of the oG. **a** Various G genes used in this study. The G gene was located at the region between the M gene and L gene in the genome of RV. The B19G is a wild type G gene of RV SAD B19 strain [3], the oG is a codon-optimized chimeric version [15], and ooG is a rearranged gene based on the mouse CPB in this study. **b** Each salmon red circle represents a CPBS of a single mouse gene plotted against its gene length. The average CPBS of mouse is 0.06508. The carolina blue dot and the violet dot represent the oG and ooG genes, respectively. The average CPBSs of the oG and ooG are − 0.0738 and 0.3114, respectively. **c** Characteristics of the oG and ooG genes. The reshuffled oG (ooG) is possible to optimize the ability of protein expression in mouse, while the oG might be deoptimized in mouse

RV has wide infection range, such as canine, mouse and human. Among these animals, canine is one of the RV reservoir hosts, which providing the opportunity that RV has adopted the CPB of canine during the long-term co-evolution. While, RV-based tools are usually used in mouse model in neuroscience research. Therefore, the codon pair score (CPS) of canine, mouse and human were calculated using previously reported method [17]. Briefly, a total of 45,094, 68,272 and 110,788 genes for canine, mouse and human were analyzed to calculate codon pair bias score for each of the 3721 codon pair (61 × 61 codons). The CPS values of these three species are very similar (Fig. 1a-c), however, the relationships of the three species are different. The correlation coefficient value of codon pair preference between human and canine (Spearman rho = 0.9859) is closer than that between mouse and canine (Spearman rho = 0.9459), and also closer than that between human and mouse (Spearman rho = 0.9588). Next, we calculated the CPBS values for each gene of mouse and canine respectively and plotted the CPBS value against its gene length. We found that the majority of genes in mouse and canine have positive CPBS, averaged at 0.0651 and 0.0704, respectively, which are similar to the human 0.0703 (Additional file 1: Fig. S1). Therefore, these data provide a basic for oG reshuffling (Fig. 2a).

### ooG has higher expression level than oG

The oG, the best version of G in current RV-based tools [15], was selected as a model for codon pair optimization based on codons pairs preference data from the mouse genome to improve its expression level. Based on the CPS obtained from the mouse, we attempted to reshuffle the oG sequence to improve oG’s expression level, and named the new oG sequence as ooG (Additional file 1: Fig. S2). The median CPBS of oG and ooG is -0.0738, 0.3114, respectively (Fig. 2b, c). The number of CpG and UpA are 73 and 45 for oG, 38 and 34 for ooG (Fig. 2c).

**Fig. 3:**
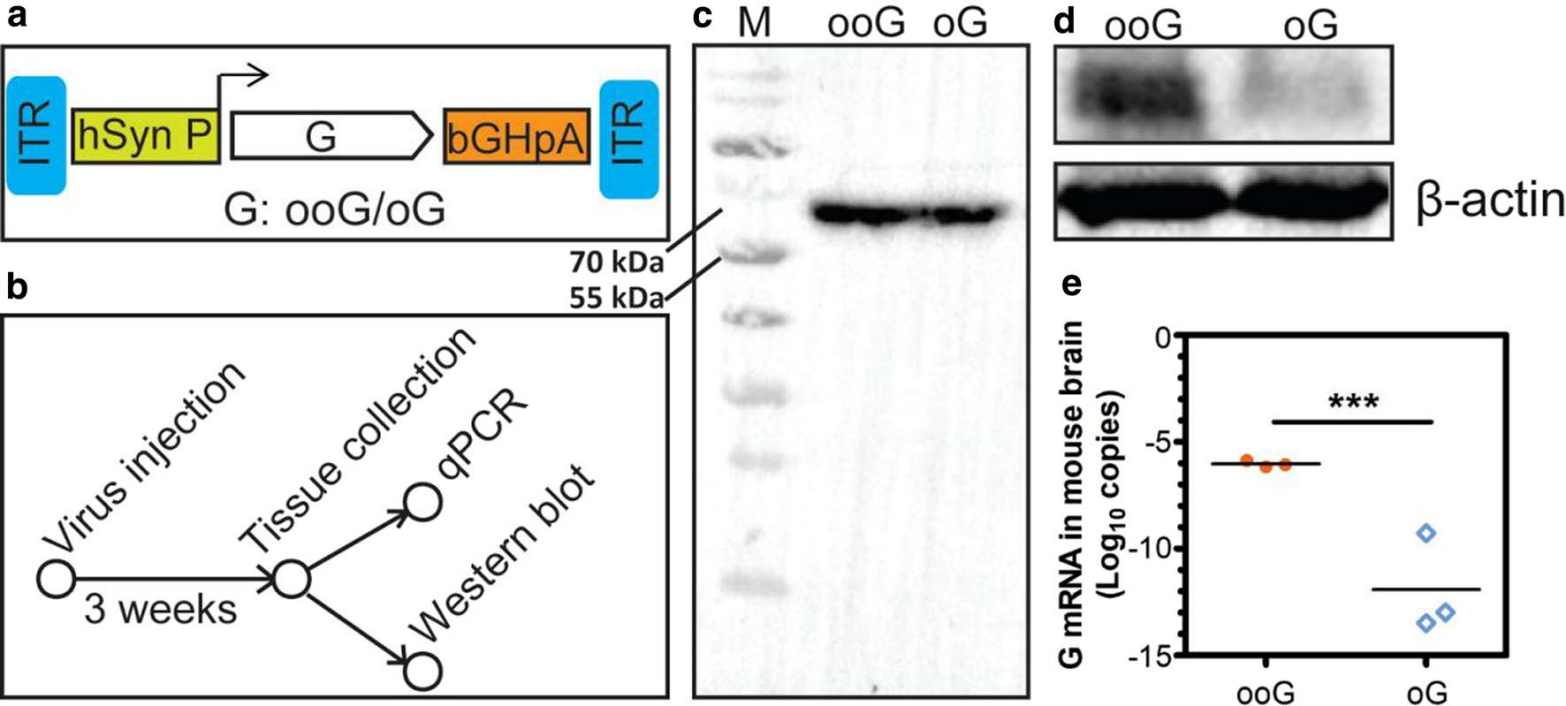
The characteristics of the ooG and oG in mouse. **a** Design of scAAV helper viruses express various RV G genes (ooG and oG). **b** The workflow of viruses labeling and sample treating. **c** The specificity of the antibody against the ooG and the oG was determined by western blot. **d** The ooG and G protein expression in mice brains were measured by Western blotting using antibody against the ooG. **e** The mRNA copies of the ooG and oG in mice brains were examined using quantitative RT-PCR. The t-test was conducted to compare the difference of mRNA copies of the ooG and oG. “***” Represents a statistically significant difference. The p-value of the ooG versus oG is 0.0004. The data is from the three independent experiments

Next, the genes of the ooG and oG were commercially synthesized and cloned into the self-complementary AAV (scAAV) vector, respectively [20], in which the ooG or oG was expressed by the syn promoter (Fig. 3a). 150 μl of scAAV-hsynP-ooG-bGHpA and scAAV-hsynP-oG-bGHpAwere injected into the ventral hippocampus (vHPC) region of C57BL/6 mouse, respectively. After three weeks, the brain tissue was collected and analyzed (Fig. 3b). Firstly, protein level was determined using antibody against G, which is specific to the ooG and the oG (Fig. 3c). We found that the amount of ooG protein is higher than oG’s (Fig. 3d). Furthermore, we found that the ooG mRNA copies are higher than oG’s (Fig. 3e). Collectively, the ooG as a novel gene has higher protein expression level than oG.

### ooG can enhance the trans-synaptic efficiency

Based on the data, we provide a hypothesis that the ooG has a potential ability of increasing RV trans-synaptic efficiency. To directly compare the transsynaptic efficiency of the ooG, oG, and B19G, the long-distance input data in known circuit were collected and analyzed by calculating the convergence index. ssAAV-EF1α-DIO-EGFP-F2A-TVA-WPRE-bGHpA was co-injected with one of the three AAVs: scAAV-hsynP-ooG-bGHpA, scAAV-hsynP-oG-bGHpAand scAAV-hsynP-B19G-bGHpA into the Vhpc region of Thy-1 cre mouse, respectively. After three weeks, the EnvA-pseudorabies (Enva-RV844) were injected into the same region. The brains were collected and processed seven days later.

**Fig. 4:**
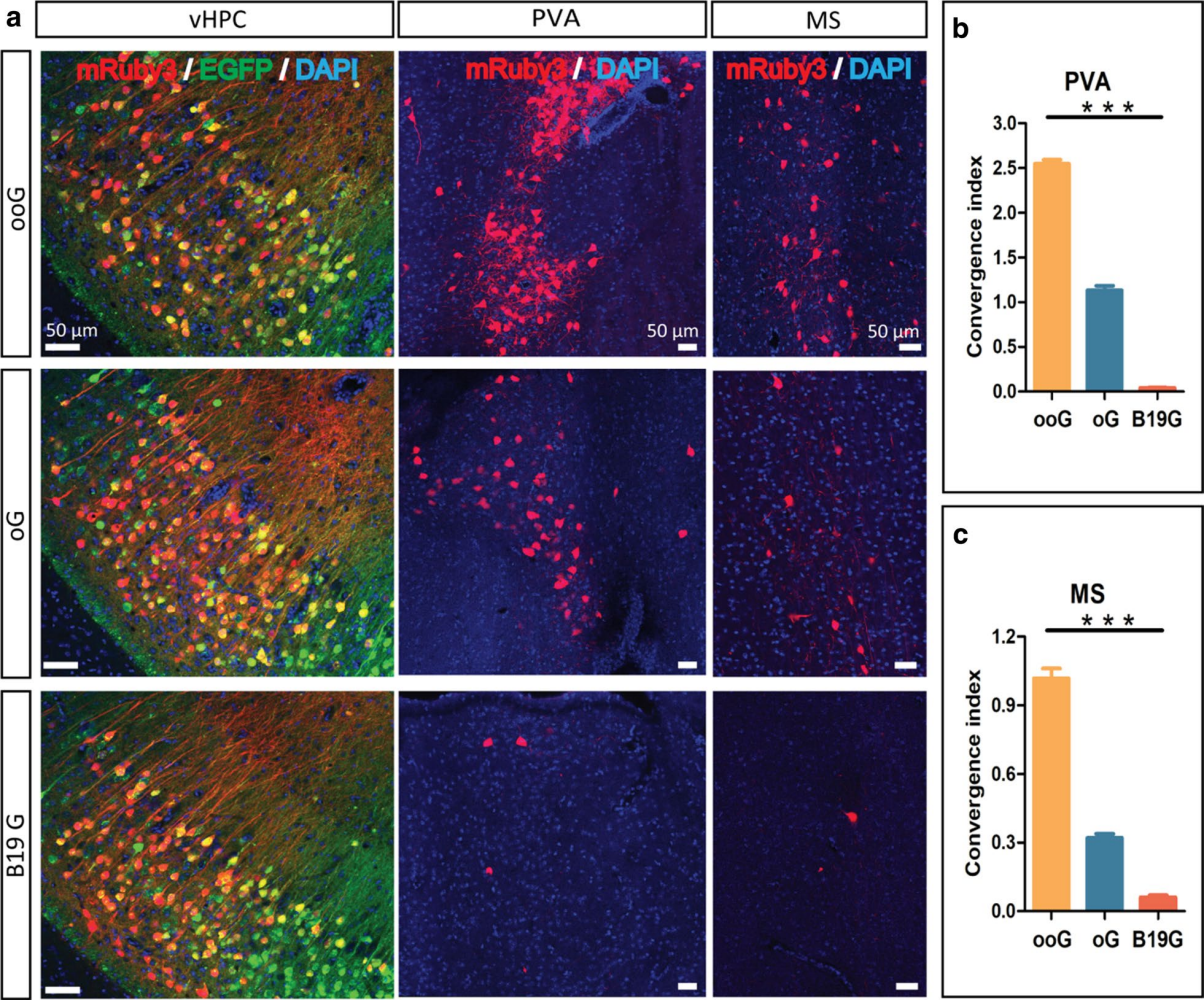
Trans-monosynaptic tracing efficiency of RV with the help of various glycoproteins (ooG, oG and B19G). **a** Coronal sections were collected and imaged. Representative images of EnvA-RV-mRuby3 (EnvA-RV844) retrogradely trans-monosynaptic tracing from the vHPC to PVA with the help of two AAV helpers. **b** and **c** Convergence indices for long-distance projection inputs in PVA (**b**) and MS (**c**) using the ooG, oG, and B19G, respectively. The one-way ANOVA was conducted to compare the difference of RV tracing efficiency using different G genes (GraphPad Prism 5 software). Three mice for each G gene. “***” Represents a statistically significant difference. Definitely, the p-values of the ooG versus oG, ooG versus B19G and oG versus B19G are less than 0.0001, respectively. Error bars indicate the standard error of the mean from the three independent experiments

**Fig. 5:**
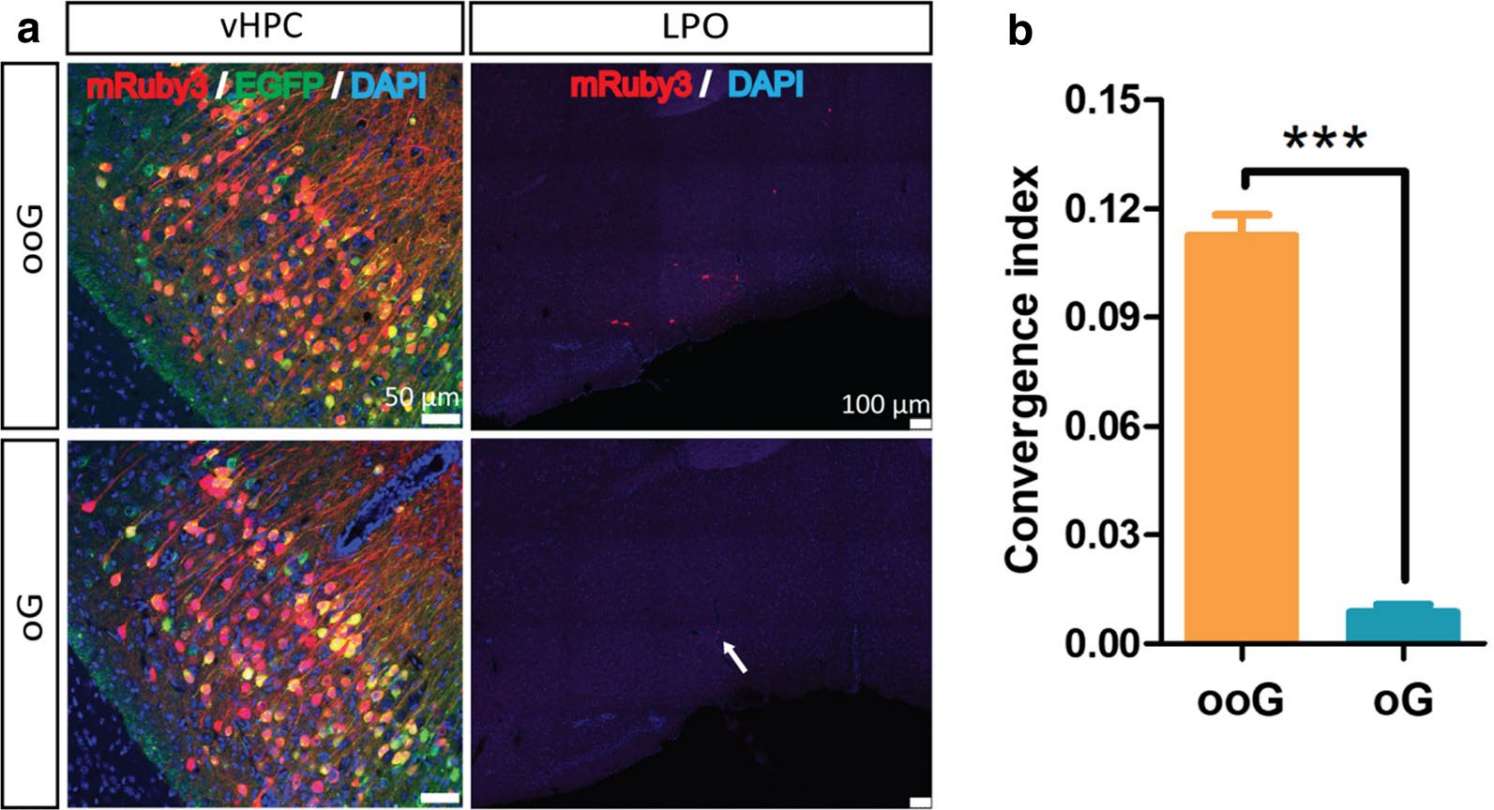
Trans-monosynaptic efficiency of RV with the help of the ooG and oG. **a** Coronal sections were collected and imaged. Representative images of Enva-RV844 retrogradely trans-monosynaptic tracing from the vHPC to LPO with the help of two AAV helpers. **b** Convergence indices for projection inputs in the LPO using ooG and oG. The t-test was conducted to compare the difference of RV tracing efficiency using different G genes. Three mice for each G gene. “***” Represents a statistically significant difference. The p-value of ooG versus oG is less than 0.0001. Error bars indicate the standard error of the mean from the three independent experiments

We observed the main long-distance input regions are the paraventricular thalamic nucleus (PVA) and the medial septal nucleus (MS), which are known and consistent with the previous reports (Fig. 4a) [21]. Then the convergence index was calculated to directly compare the retrograde trans-synaptic efficiency (14) of these three G proteins by using the number of input neurons (mRuby3 positive) divided by the number of starter cells (EGFP and mRuby3 positive). In PVA region, the convergence index is the 2.547 ± 0.05, 1.132 ± 0.05 and 0.041 ± 0.01 for ooG, oG and B19G, respectively (Fig. 4b). In MS region, the convergence index is the 1.017 ± 0.04, 0.320 ± 0.02 and 0.061 ± 0.01 for the ooG, oG and B19G, respectively (Fig. 4c). Comparison with the data from the ooG, oG and B19G, we found that the convergence index of ooG is 2.3-fold and 62.1-fold higher than the oG and B19G, and the convergence index of oG is 27.6-fold higher than the B19G in PVA region which is similar with previous report [15]. The convergence index of the ooG is 3.2-fold and 16.7-fold higher than the oG and B19G, and the convergence index of the oG is 5.2-fold higher than the B19G in MS region. In addition, the convergence index is the 0.113 ± 0.006 and 0.009 ± 0.002 for the ooG and oG in lateral preoptic area (LPO), and the convergence index of the ooG is 12.6-fold higher than the oG (Fig. 5).

In addition, we compared the effects of the ooG and oG on labeling specificity and cellular toxicity. Firstly, the whole-brain connections to the vHPC were analyzed to investigate the labeling specificity, which revealed that the same brain regions were labeled in the ooG and oG groups (Additional file 1: Fig. S3a). These results indicated that the labeling specificity of the ooG is consistent with the oG. Secondly, the cellular toxicity of the ooG and the oG were determined by detecting the caspase-3 and cell morphology. These results showed that the signals of caspase-3 is not obvious in the ooG and oG groups (Additional file 1: Fig. S3b), and the cell morphologies in ooG and oG groups are similar (Additional file 1: Fig. S3c).

## Discussion

To label the complete pre-synaptic and post-synaptic partners of a specific neuron is the ultimate goal for the neural circuit tracers. RV as the most important and popular tool has been used widely. However, the current systems can only label a fraction of presynaptic neurons [7]. Previous studies showed that the amount of the G protein is one of the key steps for adjusting the trans-synaptic efficiency [15]. Therefore, many efforts have been taken for elevating the expression level of G protein. The direct method is using the stronger promoter to enhance the protein amount. Various promoters have different ability of improving gene expression in various cell types [22, 23]. Miyamichi et al. select CAG as a stronger promoter to drive G expression [14]. Screening different G proteins from various rabies virus strains is another method for improving RV trans-synaptic tracing efficiency. Kim et al. compared various G (N2C strain and Pasteur strain) and engineered a chimeric G (oG) using codon-optimized strategy [15]. In addition, providing more copies of the G gene might be a simple method for increasing gene expression. While the package capacity of AAV is limited. Therefore, screening and optimizing G gene is an appropriate method for increasing the gene expression to improve RV trans-synaptic tracing efficiency.

During the long-term evolution, virus has adapted its host’s environment for reproducing. All mammals are susceptible to rabies virus infection, only a few species are its reservoirs. Therefore, virus gene codons and gene codon pairs are adaptive to its reservoirs. Canine is one of RV reservoirs, while mouse is not. Therefore, the replicating and packaging efficiency of RV is not optimal in mouse. Mouse is the most important animal model for neuroscience research. Reshuffling virus genes according to the mouse genetic context might provide the proper condition for virus life cycle. CPB is a stable characteristic of a species. Based on the CPB strategy, attenuated viruses have been developed using under represented codon pair to deoptimize gene in RNA viruses and DNA viruses [17–19, 24–26]. In these cases, deoptimized gene has rare codon pairs and more CpG and UpA, which might cause the lower gene expression. Several mechanism of virus attenuation were appeared, (i) under represented codon pair results in decreasing efficiency of translation, such as translation elongation rate, translation initiation complex dissociation, and protein processing [17, 19, 24, 26, 27], (ii) more CpG and UpA result in inducing an innate immune response and reducing mRNA stability [28–31]. However, the exact mechanism of virus attenuation is still disputed and needs to be determined.

Unlike these previous studies, we provided a hypothesis that optimize gene using overrepresented codon pair to improve protein expression according to the mouse genome characteristic. Indeed, the ooG protein expression amount is higher than the oG (Fig. 3d, e). Therefore, ooG can significantly increase the RV trans-synaptic efficiency (Fig. 4a–c). The characteristics of the oG and ooG in mouse are that the CPBS is − 0.0738 and 0.3114, the number of CpG is 73 and 38, and the number of UpA is 45 and 34, respectively (Fig. 2c). Based on the results and possible mechanism for virus attenuated, we proposed that the over represented codon pair ooG provides a suitable situation for elevating translation efficiency, reducing an innate immune response and helping mRNA stability. However, the real mechanism should be determined in future. Collectively, whatever the mechanism for CPB, it has become increasingly clear that CPB has a profound impact on protein expression.

In the present study, using RV as a model target and trans-synaptic tracing efficiency as a readout, we verified our hypothesis that nucleic acid sequence optimization based on CPB analysis is a convenient and efficient strategy to improve protein expression level. The ooG will be a useful tool for mapping neural circuits. Importantly, the approach reported here might provide a convenient, efficient and universal strategy to improve protein expression for various application scenarios such as other tracers, cell engineering, vaccine and oncolytic virus designs.

## Materials and methods

### Animals

All procedures were approved by the Animal Care and Use Committees at the Shenzhen Institute of Advanced Technology or Innovation Academy for Precision Measurement Science and Technology, Chinese Academy of Sciences. Eight-week-old male C57BL/6 mice were used for testing the protein expression and mRNA level. Eight-week-old Thy1-Cre mice were used for analyzing the retrograde trans-synaptic efficiency of rabies virus with the trans-complementary with various version G proteins, which expresses Cre in the nervous system of transgenic mice.

### Rearrangement of the oG

Firstly, the codon pair bias of mouse, canine and human were calculated using the previous method [17] based on the gene CDS annotation of each species from the Ensembl database release 98, which were based on the genome assembly GRCm38, CanFam3.1 and GRCh38.p13, respectively. Secondly, the oG gene was optimized based on the mouse CPS according to the general rule of maintaining the mean free energy of the RNA folding within a suitable range and to avoid dramatic changes in its secondary structure.

The algorithm to calculate CPS and CPBS for coding sequence was developed after the description of Kunec et al. [16] and Coleman et al. [17]. For a specific codon pair, the CPS is defined as the natural logarithm of the result of the observed codon pairs number divided by the expected number of codon pairs in all coding sequences of a species. The observed number is the actual number of the codon pair in the coding sequences. And the expected number of a codon pair is a theoretical value based on the proportion of amino acid and codon of the codon pair.

$$CPS = \ln \left( {\frac{{Obs}}{{Exp}}} \right)$$

where

$$Exp = \frac{{N(codon_{1} ) \times N(codon_{2} )}}{{N(aa_{1} ) \times N(aa_{2} )}} \times N(aa_{1} aa_{2} )$$

The *N*(*codon*_1_) and *N*(*codon*_2_) denote the number of occurrences of two codons in a codon pair, respectively. The *N*(*aa*_1_) and *N*(*aa*_2_) are the number of corresponding amino acids. And *N*(*aa*_1_*aa*_2_) is the number of the amino acid pair for the codon pair above. A positive CPS suggested the codon pair is over represented in the species, whereas a negative value is under represented.

Then, the CPBS for a coding sequence is the result of the arithmetic mean of all codon pair CPS values.

$$CPBS = \sum\limits_{{i = n}}^{n} {\frac{{CPS_{i} }}{{n - 1}}}$$

### Preparation AAV and RV

scAAV-hsynP-ooG-bGHpA, scAAV-hsynP-oG-bGHpA, and scAAV-hsynP-B19G-bGHpA were constructed by inserting the expression cassette into the self-complementary AAV (scAAV) core vector [20]. ssAAV-EF1α-ooG-WPRE-bGHpA and ssAAV-EF1α-DIO-EGFP-F2A-TVA-WPRE-bGHpA were constructed by inserting the expression cassette into the single-strand AAV2 core vector (addgene 20298). All plasmids were confirmed by sequencing. Then these recombinant AAVs were packaged with the AAV9 capsid, respectively.

Previous report shows that the mRuby3 is a new version red fluorescent protein with high lighter and stabilization [32]. Therefore, the mRuby3 gene was selected and inserted into pSADdeltaG-F3 vector [33]. Then the plasmid was confirmed by sequencing. The EnvA-RV-mRuby3 (EnvA-RV844) was prepared using the previous method [2].

### Stereotaxic microinjection in mice brains

Briefly, mice were anesthetized with chloral hydrate before injection. scAAV-hsynP-ooG-bGHpA (6.9 × 10^11^ vg/ml), scAAV-hsynP-oG-bGHpA (1.1 × 10^12^ vg/ml) and scAAV-hsynP-B19G-bGHpA (2 × 10^12^ vg/ml) were respectively mixed (1:1, total volume 80 nl) with ssAAV-EF1α-DIO-EGFP-F2A-TVA-WPRE-bGHpA (6.2 × 10^11^ vg/ml) and co-injected into the vHPC region of mice. Then, the EnvA-RV-mRuby3 (EnvA-RV844, 2 × 10^8^ FFU/ml) was injected into the same region after three weeks. During the process of virus injection, syringes were left in place for 10 min following injections to minimize diffusion. All surgical mice were back to the animal facility for extra seven days for fluorescent protein expression and RV trans-synaptic spreading.

### Brain section and immunohistochemistry

Brain sections were collected referred in previous studies [13]. Briefly, mice were anaesthetized and transcardially perfused with 0.9% saline followed by 4% paraformaldehyde solution. The brains were removed and post-fixed over-night in 4% paraformaldehyde before being sectioned into 50 µm slices, and slices were stained with DAPI. For staining the caspase-3, fixed slices were immunostained with the caspase-3 antibody (1:500, Cell Signaling, #9661) and amplified with the goat anti-rabbit secondary antibody (1:500, Jackson, #611-605-215), and slices were stained with DAPI. Slices were imaged using the Leica TCS SP8 confocal microscope or the Olympus VS 120 slide scanning system.

### Preparation of antibody against the ooG

BALB/c mice were immunized directly by intramuscular injection of 200 μl of AAV mixture with phosphate buffered saline, which contains 20 μl of ssAAV-EF1α-ooG-WPRE-bGHpA (1.4 × 10^12^ vg/ml). Two weeks later, the second immunization was performed by intramuscular injection of the same amount AAV and the sera were collected after two weeks. Then, the specificity of the antibody was confirmed by using western blot to detect the expression of the ooG and the oG in the 293 T cells, which were separately transfected with 2 μg of plasmids containing the expression cassettes of the ooG (pAAV-Ef1a-ooG-WPRE-BGHpA) and the oG (pAAV-Ef1a-oG-WPRE-BGHpA).

### Western blot

150 nl of scAAV-hsynP-ooG-bGHpA (8.7 × 10^11^ vg/ml) and scAAV-hsynP-oG-bGHpA (1.3 × 10^12^ vg/ml) were injected into the vHPC region of C57BL/6 mouse, respectively. After three weeks, the tissue was collected and treated, one part for RNA extraction, and another for western blot. The tissue lysate or 293 T cells were separately analyzed on a 12% SDS-PAGE, and then electro-transferred to PVDF Immobilon-P membranes (Millipore), blocked with 5% skim milk in TBST, and then treated with antibody against ooG at 1:500 dilution for 1 h at room temperature. After washing 3 times in TBST, the secondary anti-mouse antibody conjugated to Horseradish peroxidase at 1:5000 dilution was applied to the blots for 1 h at room temperature. To quantify the expression amount of GAPDH, the protein was analyzed by using fisrt antibody (1:2000) and second antibody (1:5000). Signal was detected with ECL western blotting reagent.

### Quantitative real-time PCR

Total RNA was extracted from tissue using TRIzol Reagent. DNA was digested using DNaseI for 30 min at 37 °C. 1 μg of RNA was reverse transcribed into cDNA using random hexamers. Specific primers (for ooG, forward primer, GGCCTACAACTGGAAGATGGC, for oG, forward primer, AGCTTACAACTGGAAGATGGC, and they have the same reverse primer, TAGAAGACACCGCTACTCCT, for GAPDH, forward primer, GGTGAAGGTCGGTGTGAACG, reverse primer, CTCGCTCCTGGAAGATGGTG) were used to quantify by qPCR using the SYBR Green Master Mix on the real-time PCR detection system (Bio-Rad). The copy numbers were determined based on standard curve generated for each gene using known concentration plasmids pSyn-ooG, pSyn-oG, and pcDNA-GAPDH.

## Supplementary Information

**Additional file 1: Fig. S1.** Distribution of codon pair bias scores in human, canine and mouse. CPBSs were calculated using the previous described method [17], Each salmon red circle represents a CPBS of a single human (a), canine (b), and mouse (c) gene plotted against its gene length, respectively. The average CPBSs of the human, canine and mouse are 0.0703, 0.0704, and 0.0651, respectively. **Fig. S2.** The sequence of the ooG. The ooG sequence was produced based on the oG [15] by using a previous described method [17]. > ooG sequence ATGGTTCCTCAAGCCCTTCTCTTTGTTCCTCTTCTTGTCTTCCCGCTCTGCTTTGGGAAGTTCCCCATCTACACCATTCCTGACAAGCTAGGGCCCTGGAGCCCCATTGACATCCACCACCTCAGCTGCCCCAACAACTTGGTTGTAGAAGATGAAGGCTGCACCAACCTCAGTGGCTTCTCCTACATGGAGCTAAAAGTGGGCTACATCTCGGCCATCAAGATGAATGGCTTCACCTGCACTGGAGTTGTCACTGAAGCAGAGACCTACACCAACTTTGTTGGCTATGTCACCACCACCTTCAAAAGAAAACACTTCCGGCCCACTCCAGATGCCTGCCGCGCGGCCTACAACTGGAAGATGGCGGGGGACCCCCGCTATGAAGAGAGCCTGCACAACCCCTACCCAGACTACCACTGGCTGAGGACTGTGAAGACCACCAAAGAAAGTTTGGTCATCATCAGCCCCAGTGTAGCTGACTTGGACCCCTATGACCGTTCTCTACACAGCCCTGTATTTCCTGGTGGGAACTGCAGTGGTGTGGCTGTCAGCAGCACCTACTGCAGCACCAACCATGACTACACCATCTGGATGCCGGAGAACCCCCGGCTAGGGATGTCCTGTGACATCTTCACCAACAGCCGAGGGAAAAGAGCCAGCAAAGGTTCTGAGACCTGTGGCTTTGTAGATGAGCGTGGCCTCTACAAGAGTTTAAAAGGTGCCTGCAAATTAAAACTCTGTGGTGTTCTTGGTCTTCGGCTCATGGATGGCACCTGGGTGGCCATGCAGACCAGCAATGAGACCAAGTGGTGCCCGCCGGGCCAGCTTGTCAACCTCCATGACTTCCGAAGTGATGAAATAGAACATCTTGTTGTAGAAGAACTTGTCAAGAAAAGAGAAGAATGTTTAGATGCCCTGGAGAGCATCATGACTACCAAGAGTGTCTCCTTCCGTCGCCTCAGCCACCTCAGGAAACTTGTTCCTGGCTTTGGGAAAGCCTACACCATCTTCAACAAGACGCTCATGGAAGCAGATGCCCACTACAAATCTGTCCGCACGTGGAATGAGATCATTCCTTCCAAAGGCTGCCTCCGAGTTGGTGGCCGCTGCCACCCACATGTCAATGGTGTCTTCTTCAATGGCATCATTCTTGGGCCAGATGGAAATGTCCTCATTCCAGAGATGCAGAGCAGCCTGCTGCAGCAGCACATGGAACTTCTTGTCAGCAGTGTCATCCCGCTCATGCACCCGCTGGCAGACCCCAGCACTGTCTTCAAGAATGGAGATGAAGCAGAAGATTTTGTAGAAGTTCATCTTCCTGATGTTCATGAAAGAATTTCTGGTGTGGACTTGGGTCTTCCCAACTGGGGAAAATATGTTCTTCTTTCTGCTGGGGCGCTCACGGCGCTCATGTTAATAATATTCCTCATGACCTGCTGcAGAAGAGTCAACCGCTCGGAGCCCACCCAGCACAACCTTCGTGGCACGGGCCGAGAAGTTTCTGTCACGCCGCAGAGTGGGAAGATCATCTCCTCCTGGGAGAGCCACAAGTCAGGAGGAGAGACGCGCCTGTAA. **Fig. S3.** The effects of the ooG on labeling specificity and cellular toxicity. (a) The percentage of input neurons from each site to total quantified inputs in the ooG and oG groups. (b) The signals of the caspase-3 was determined by immunohistochemistry using antibody against the caspase-3 in the ooG and oG groups. (b) The fine structure of the neuron is apparent in the ooG and oG groups. These images are the representatives from three mice.

## Data Availability

The data that used in this study are available from the corresponding author upon reasonable request.
