# Supplementary material for: Development of a rabies virus-based retrograde tracer with high trans-monosynaptic efficiency by reshuffling glycoprotein
Source: Mol Brain. 2021 Jul 8;14:109. doi: 10.1186/s13041-021-00821-7 (PMC8265122; doi:10.1186/s13041-021-00821-7)

**Fig. S1.** Distribution of codon pair bias scores in human, canine and mouse. CPBSs were calculated using the previous described method [17], Each salmon red circle represents a CPBS of a single human (a), canine (b), and mouse (c) gene plotted against its gene length, respectively. The average CPBSs of the human, canine and mouse are 0.0703, 0.0704, and 0.0651, respectively.


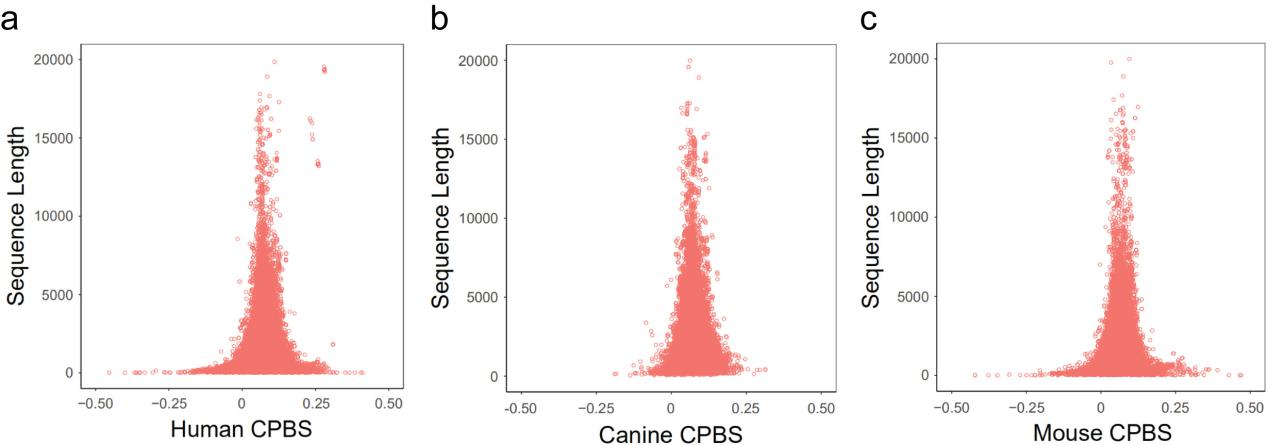


**Fig. S2.** The sequence of the ooG. The ooG sequence was produced based on the oG [15] by using a previous described method [17].

>ooG sequence

ATGGTTCCTCAAGCCCTTCTCTTTGTTCCTCTTCTTGTCTTCCCGCTCTGCTTTGGGAAGTTCCCCATCTACACCATTCCTGACAAGCTAGGGCCCTGGAGCCCCATTGACATCCACCACCTCAGCTGCCCCAACAACTTGGTTGTAGAAGATGAAGGCTGCACCAACCTCAGTGGCTTCTCCTACATGGAGCTAAAAGTGGGCTACATCTCGGCCATCAAGATGAATGGCTTCACCTGCACTGGAGTTGTCACTGAAGCAGAGACCTACACCAACTTTGTTGGCTATGTCACCACCACCTTCAAAAGAAAACACTTCCGGCCCACTCCAGATGCCTGCCGCGCGGCCTACAACTGGAAGATGGCGGGGGACCCCCGCTATGAAGAGAGCCTGCACAACCCCTACCCAGACTACCACTGGCTGAGGACTGTGAAGACCACCAAAGAAAGTTTGGTCATCATCAGCCCCAGTGTAGCTGACTTGGACCCCTATGACCGTTCTCTACACAGCCCTGTATTTCCTGGTGGGAACTGCAGTGGTGTGGCTGTCAGCAGCACCTACTGCAGCACCAACCATGACTACACCATCTGGATGCCGGAGAACCCCCGGCTAGGGATGTCCTGTGACATCTTCACCAACAGCCGAGGGAAAAGAGCCAGCAAAGGTTCTGAGACCTGTGGCTTTGTAGATGAGCGTGGCCTCTACAAGAGTTTAAAAGGTGCCTGCAAATTAAAACTCTGTGGTGTTCTTGGTCTTCGGCTCATGGATGGCACCTGGGTGGCCATGCAGACCAGCAATGAGACCAAGTGGTGCCCGCCGGGCCAGCTTGTCAACCTCCATGACTTCCGAAGTGATGAAATAGAACATCTTGTTGTAGAAGAACTTGTCAAGAAAAGAGAAGAATGTTTAGATGCCCTGGAGAGCATCATGACTACCAAGAGTGTCTCCTTCCGTCGCCTCAGCCACCTCAGGAAACTTGTTCCTGGCTTTGGGAAAGCCTACACCATCTTCAACAAGACGCTCATGGAAGCAGATGCCCACTACAAATCTGTCCGCACGTGGAATGAGATCATTCCTTCCAAAGGCTGCCTCCGAGTTGGTGGCCGCTGCCACCCACATGTCAATGGTGTCTTCTTCAATGGCATCATTCTTGGGCCAGATGGAAATGTCCTCATTCCAGAGATGCAGAGCAGCCTGCTGCAGCAGCACATGGAACTTCTTGTCAGCAGTGTCATCCCGCTCATGCACCCGCTGGCAGACCCCAGCACTGTCTTCAAGAATGGAGATGAAGCAGAAGATTTTGTAGAAGTTCATCTTCCTGATGTTCATGAAAGAATTTCTGGTGTGGACTTGGGTCTTCCCAACTGGGGAAAATATGTTCTTCTTTCTGCTGGGGCGCTCACGGCGCTCATGTTAATAATATTCCTCATGACCTGCTGcAGAAGAGTCAACCGCTCGGAGCCCACCCAGCACAACCTTCGTGGCACGGGCCGAGAAGTTTCTGTCACGCCGCAGAGTGGGAAGATCATCTCCTCCTGGGAGAGCCACAAGTCAGGAGGAGAGACGCGCCTGTAA

**Fig. S3.** The effects of the ooG on labeling specificity and cellular toxicity. (a) The percentage of input neurons from each site to total quantified inputs in the ooG and oG groups. (b) The signals of the caspase-3 was determined by immunohistochemistry using antibody against the caspase-3 in the ooG and oG groups. (b) The fine structure of the neuron is apparent in the ooG and oG groups. These images are the representatives from three mice.


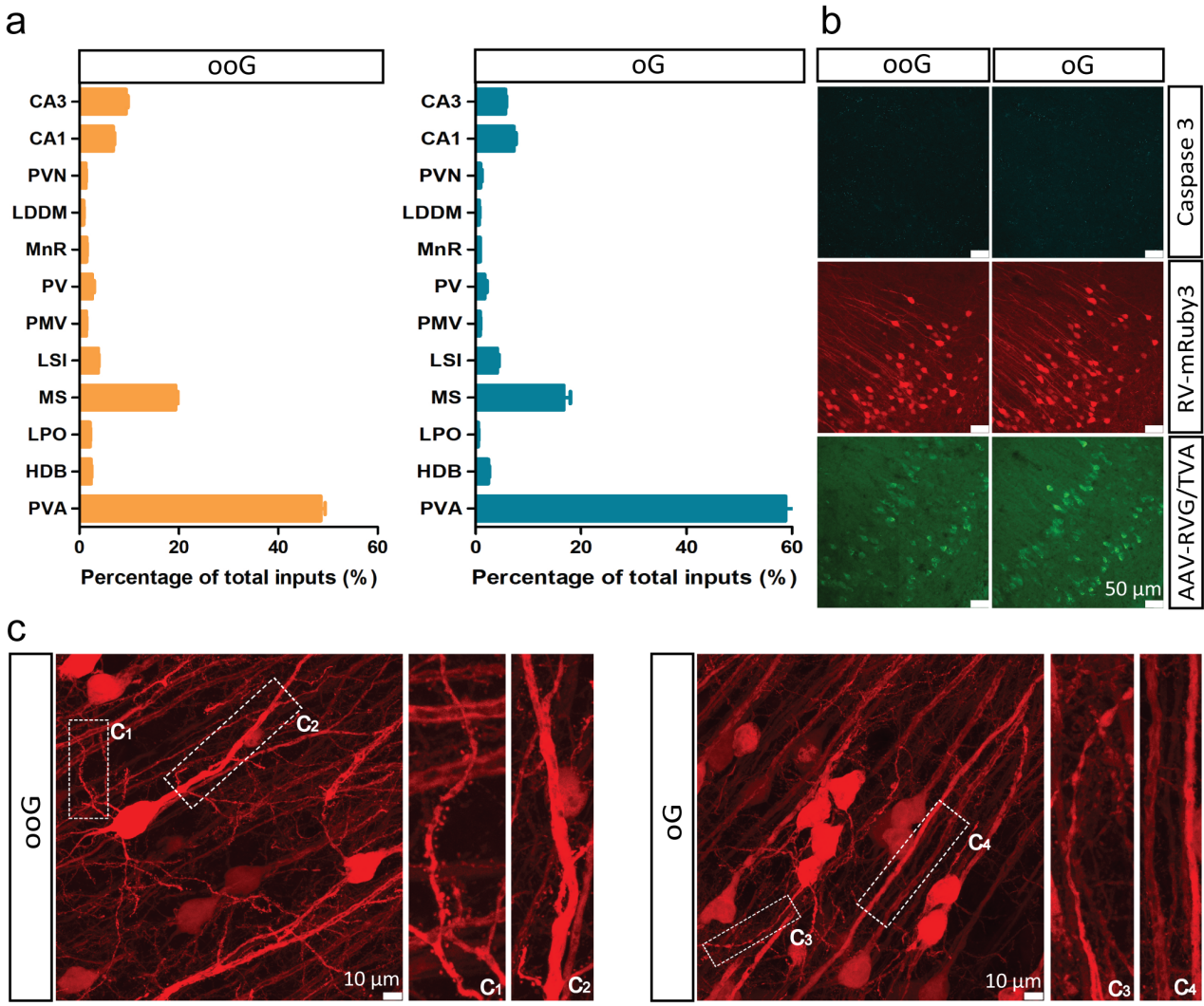

Supplement: Supplementary file 1 — Additional file 1: Fig. S1. Distribution of codon pair bias scores in human, canine and mouse. CPBSs were calculated using the previous described method [17], Each salmon red circle represents a CPBS of a single human (a), canine (b), and mouse (c) gene plotted against its gene length, respectively. The average CPBSs of the human, canine and mouse are 0.0703, 0.0704, and 0.0651, respectively. Fig. S2. The sequence of the ooG. The ooG sequence was produced based on the oG [15] by using a previous described method [17]. > ooG sequence ATGGTTCCTCAAGCCCTTCTCTTTGTTCCTCTTCTTGTCTTCCCGCTCTGCTTTGGGAAGTTCCCCATCTACACCATTCCTGACAAGCTAGGGCCCTGGAGCCCCATTGACATCCACCACCTCAGCTGCCCCAACAACTTGGTTGTAGAAGATGAAGGCTGCACCAACCTCAGTGGCTTCTCCTACATGGAGCTAAAAGTGGGCTACATCTCGGCCATCAAGATGAATGGCTTCACCTGCACTGGAGTTGTCACTGAAGCAGAGACCTACACCAACTTTGTTGGCTATGTCACCACCACCTTCAAAAGAAAACACTTCCGGCCCACTCCAGATGCCTGCCGCGCGGCCTACAACTGGAAGATGGCGGGGGACCCCCGCTATGAAGAGAGCCTGCACAACCCCTACCCAGACTACCACTGGCTGAGGACTGTGAAGACCACCAAAGAAAGTTTGGTCATCATCAGCCCCAGTGTAGCTGACTTGGACCCCTATGACCGTTCTCTACACAGCCCTGTATTTCCTGGTGGGAACTGCAGTGGTGTGGCTGTCAGCAGCACCTACTGCAGCACCAACCATGACTACACCATCTGGATGCCGGAGAACCCCCGGCTAGGGATGTCCTGTGACATCTTCACCAACAGCCGAGGGAAAAGAGCCAGCAAAGGTTCTGAGACCTGTGGCTTTGTAGATGAGCGTGGCCTCTACAAGAGTTTAAAAGGTGCCTGCAAATTAAAACTCTGTGGTGTTCTTGGTCTTCGGCTCATGGATGGCACCTGGGTGGCCATGCAGACCAGCAATGAGACCAAGTGGTGCCCGCCGGGCCAGCTTGTCAACCTCCATGACTTCCGAAGTGATGAAATAGAACATCTTGTTGTAGAAGAACTTGTCAAGAAAAGAGAAGAATGTTTAGATGCCCTGGAGAGCATCATGACTACCAAGAGTGTCTCCTTCCGTCGCCTCAGCCACCTCAGGAAACTTGTTCCTGGCTTTGGGAAAGCCTACACCATCTTCAACAAGACGCTCATGGAAGCAGATGCCCACTACAAATCTGTCCGCACGTGGAATGAGATCATTCCTTCCAAAGGCTGCCTCCGAGTTGGTGGCCGCTGCCACCCACATGTCAATGGTGTCTTCTTCAATGGCATCATTCTTGGGCCAGATGGAAATGTCCTCATTCCAGAGATGCAGAGCAGCCTGCTGCAGCAGCACATGGAACTTCTTGTCAGCAGTGTCATCCCGCTCATGCACCCGCTGGCAGACCCCAGCACTGTCTTCAAGAATGGAGATGAAGCAGAAGATTTTGTAGAAGTTCATCTTCCTGATGTTCATGAAAGAATTTCTGGTGTGGACTTGGGTCTTCCCAACTGGGGAAAATATGTTCTTCTTTCTGCTGGGGCGCTCACGGCGCTCATGTTAATAATATTCCTCATGACCTGCTGcAGAAGAGTCAACCGCTCGGAG [file 13041_2021_821_MOESM1_ESM.docx]
